# Bilateral cervical chondrocutaneous branchial remnants in a child: a case report and review of the literature

**DOI:** 10.3389/fped.2026.1817692

**Published:** 2026-05-28

**Authors:** Abdullah Alshehri, Omar Alshenawy

**Affiliations:** Division of Pediatric Surgery, Department of Surgery, College of Medicine, King Saud University, Riyadh, Saudi Arabia

**Keywords:** branchial region, cartilage, chondrocutaneous, congenital abnormalities, excision

## Abstract

Bilateral chondrocutaneous branchial remnants (CCBRs) are rare congenital anomalies arising from the branchial apparatus. Given that they may be associated with other congenital anomalies, thorough physical examination and targeted investigations are recommended. Although typically asymptomatic, early intervention is favored for optimal cosmetic results and psychosocial well-being. Definitive management involves complete excision of both the cutaneous lesion and its underlying cartilaginous component. We present the case of a 10-year-old with bilateral neck masses, one of which appeared as a pedunculated skin tag, subsequently diagnosed as bilateral CCBRs. Following laboratory and imaging evaluation that revealed no associated anomalies, the patient underwent successful bilateral simple excision. This case highlights the importance of careful preoperative assessment to rule out potential systemic comorbidities, despite the straightforward nature of surgical treatment.

## Introduction

Cervical chondrocutaneous branchial remnants (CCBRs), initially described as accessory auricles, chondromatas, hamartomata, or appendages, are uncommon congenital lesions first described by Birkett et al. (1). The term CCBR was later designated by Atlan et al. (2). These lesions typically present as painless masses located in the lower third of the lateral neck, anterior to the sternocleidomastoid muscle, and contain a central cartilage core. Bilateral presentation is particularly unusual, with only 41 cases found in the literature. Given that CCBRs can be associated with other anomalies—including cardiovascular, genitourinary, gastrointestinal, visual, and auricular abnormalities (3–5)—systemic evaluation is recommended. Once the diagnosis is confirmed, management generally consists of simple excision of the cutaneous lesion and its underlying cartilage.

Herein, we report a case of a 10-year-old boy with bilateral CCBRs, along with a brief review of the relevant literature.

## Case presentation

Written informed consent for publication, including photographs, was obtained from the patient's parents. A 10-year-old boy presented to the pediatric surgery clinic with a skin tag on the left side of the neck present since birth. According to the mother, the lesion had slightly increased in size over time but remained painless, without discharge, swelling, or redness. The patient had no other medical problems.

**Figure 1:**
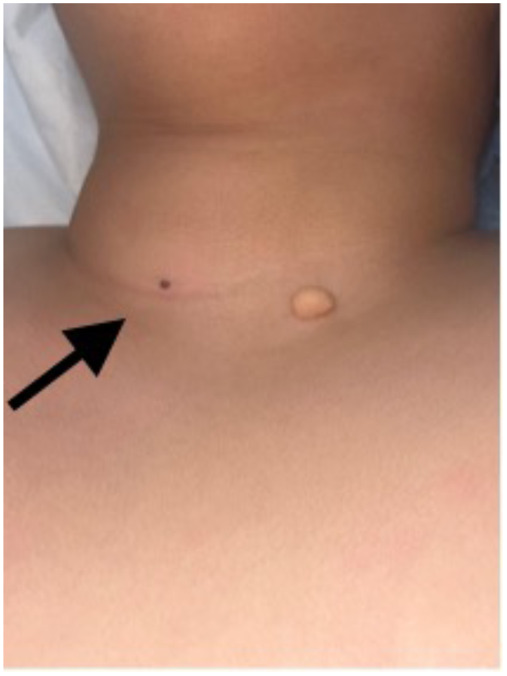
Preoperative photograph showing a skin tag on the left anterior neck. The site of the right-sided cervical chondrocutaneous branchial remnant (CCBR) is marked.

On physical examination (Figure 1), the left anterior neck exhibited a rubbery, pedunculated, oval-shaped mass measuring approximately 0.5 × 1 cm. On the right anterior neck, a small, firm mass was palpable beneath normal overlying skin, consistent with a cartilaginous remnant. The surrounding skin appeared normal, without swelling, tenderness, discharge or openings.

Routine laboratory findings were within normal limits. Focused grayscale and color Doppler ultrasonography of the anterior lower neck revealed a well-defined, homogeneous, hypoechoic exophytic oval-shaped lesion arising from the skin, measuring 1.2 × 0.6 cm, without intimal vascularity or deep extension into the subcutaneous tissue. No abnormal lymph nodes or fluid collections were identified. Renal ultrasonography, performed to screen for associated anomalies, showed normal kidneys without evidence of stone formation, calcification, or hydronephrosis. No associated anomalies were identified clinically or radiographically.

**Figure 2:**
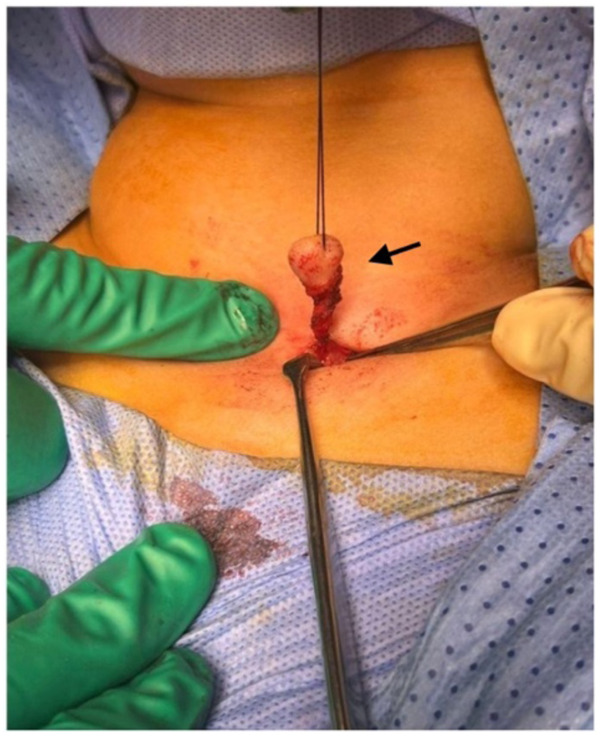
Intraoperative findings demonstrating the left-sided CCBR prior to excision.

**Figure 3:**
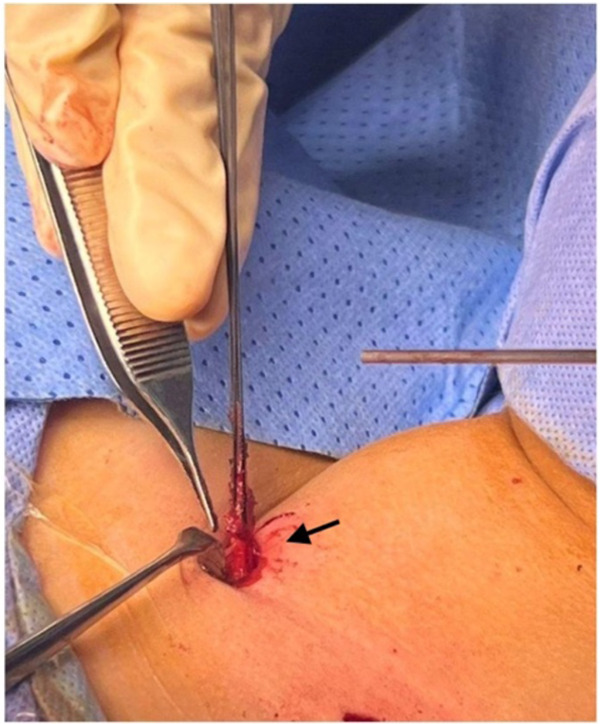
Intraoperative findings demonstrating the right-sided CCBR prior to excision.

**Figure 4:**
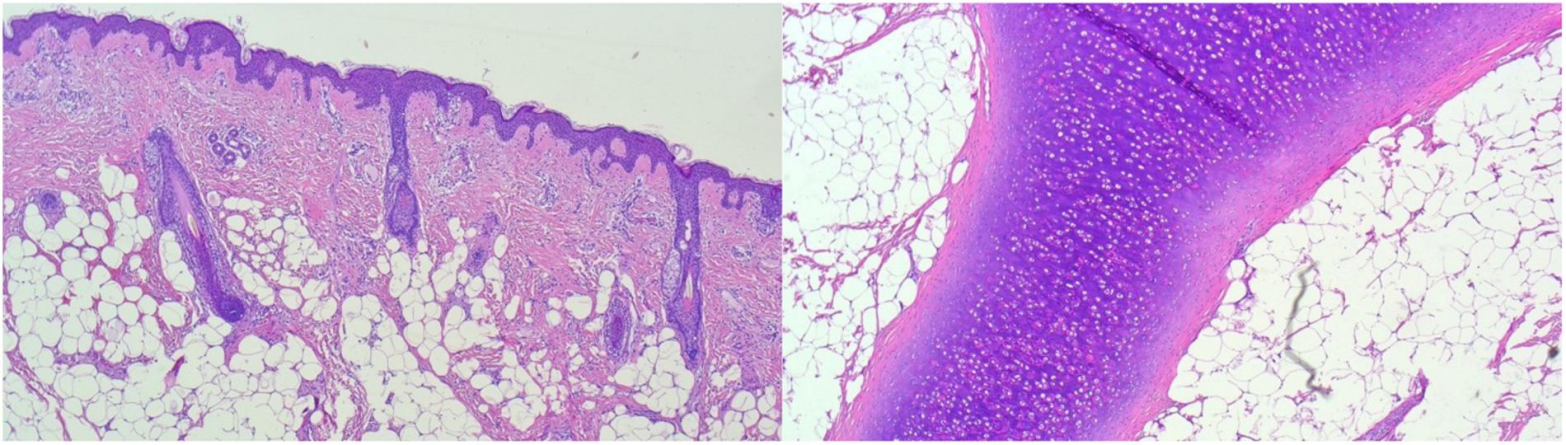
Histopathologic images of the excised lesion. Normal epidermis and dermis with adnexal structures and underlying subcutaneous fat are shown (left). A central focus of well-developed hyaline cartilage is present (right).

Bilateral CCBRs were suspected clinically and radiologically, and the patient underwent bilateral surgical excision without complications. The procedure was performed under general anesthesia. Elliptical skin incisions were made around each lesion. Careful dissection was carried down through the subcutaneous tissue. Both lesions contained cartilaginous remnants that terminated superficially at the sternocleidomastoid muscle (Figures 2, 3). Histopathological examination of the excised specimen revealed a firm, rubbery, tan-white, polypoid nodule measuring 1.5 × 1.0 × 0.5 cm. There was a central core of glistening, firm, translucent, and grey-white cartilaginous tissue. Microscopic evaluation revealed a well-developed hyaline cartilage centrally, covered by normal epidermis and dermis containing adnexal structures and subcutaneous fat—confirming the diagnosis of CCBRs (Figure 4).

**Figure 5:**
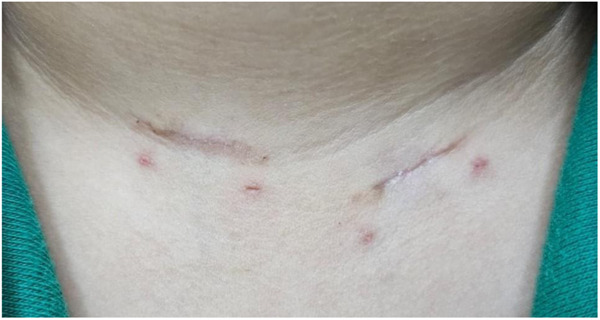
Well-healed surgical scar at the 2-week postoperative follow-up.

At the 2-week postoperative follow-up, the surgical sites were well-healed, with no complications (Figure 5).

## Discussion

CCBRs are rare congenital anomalies derived from the branchial apparatus, with only approximately 41 bilateral cases reported to date (3, 5–7). Atlan et al. reported that these lesions typically present as firm, painless nodules along the anterior border of the sternocleidomastoid muscle. Their exact embryological origin remains debated. It was suggested to either develop from ectopic auricular tissue, or from branchial tissue (2, 8). The presence of elastic cartilage suggests an origin from ectopic auricular tissue arising from the first or second branchial arches, whereas hyaline cartilage—as identified in our case—supports a cervical origin (2, 5, 8, 9). The histopathologic findings supports classifying those lesions as choristomas, defined as normal tissue located in an abnormal anatomic site (10).

The differential diagnosis of CCBRs includes branchial cleft anomalies, accessory tragus, epidermoid cysts, dermoid cysts, thyroid duct cysts, and lymphadenopathy. Clinically, CCBRs are distinguished by their firm consistency and characteristic location along the anterior border of the sternocleidomastoid muscle. Intraoperatively, the presence of a well-defined cartilaginous core without a sinus tract helps exclude branchial cleft anomalies. Histologically, the identification of mature cartilage beneath normal skin with adnexal structures confirms the diagnosis and differentiates these lesions from other cutaneous or subcutaneous masses (2, 3, 10).

Among the reported 41 cases of bilateral CCBR, six presented with associated anomalies such as ventricular septal defect, microtia, preauricular sinus or fistula, conductive hearing loss, and left superior vena cava draining into the coronary sinus (3, 5, 6, 13–16). Several studies have reported anomalies associated with unilateral CCBR. Ishigaki et al. documented 29 unilateral cases, of which eight had associated conditions, including unilateral facial paralysis, penis palmatus, bilateral undescended testes, umbilical hernia, internal strabismus, meningeal cysts, and cervical cyst (8). Begovic et al. identified 17 unilateral cases, five of which involved anomalies including branchio-oto-renal syndrome, vesicoureteral reflux, and ventricular septal defect (11). Although several studies highlight the importance of evaluating for systemic abnormalities (2, 3, 8), there are currently no standardized screening guidelines. Consistent with previously described cases of isolated bilateral CCBRs, our patient exhibited no associated anomalies in both the physical and imaging findings.

CCBRs usually present as small, asymptomatic nodules or skin tags located along the anterior border of the sternocleidomastoid muscle, growing slowly or remaining stable over time (3, 4). In our case, the left-sided lesion was clinically prominent, whereas the right-sided mass was palpable on meticulous examination, emphasizing the importance of a thorough physical examination. Ultrasonography is a useful and noninvasive tool that assists in determining lesion composition and depth while excluding vascular involvement (3, 8). Definitive diagnosis requires histopathological confirmation, typically demonstrating mature hyaline or elastic cartilage beneath normal skin with adnexal elements (2, 6, 8). Complete surgical excision of both the cutaneous and cartilaginous components remains the treatment of choice, resulting in excellent cosmetic outcomes and minimal risk of recurrence (2, 5, 6, 12). Early intervention may also help avoid psychosocial distress during childhood (6, 9, 11).

Although CCBRs are rare congenital anomalies, they can serve as external indicators to underlying developmental anomalies. Although typically benign and asymptomatic, bilateral presentations remain uncommon and warrant thorough systemic evaluation to exclude associated anomalies. Careful clinical assessment, including contralateral neck examination and appropriate imaging, is essential for an accurate diagnosis. In the presence of associated anomalies, genetic evaluation should be considered to assess for underlying syndromic conditions. Simple surgical excision provides definitive treatment with excellent cosmetic results. Awareness of this condition among pediatric surgeons and other healthcare providers facilitates early recognition, comprehensive assessment, and timely management to ensure optimal functional and psychosocial outcomes among children.

## Data Availability

The raw data supporting the conclusions of this article will be made available by the authors, without undue reservation.
